# Effectiveness Of The Core Activation And Rehabilitation Exercises For Knee Osteoarthritis - Program (CARE -KOA ^©^) Among Patients Diagnosed With Knee Osteoarthritis.

**DOI:** 10.12688/f1000research.163321.2

**Published:** 2025-06-11

**Authors:** Dias Tina Thomas, Charu Eapen, Atmananda S Hegde, Ajit R. Mahale, Prajwal Prabhudev Mane, Saurabh Mehta

**Affiliations:** 1 Department of Physiotherapy, Kasturba Medical College Mangalore, Manipal Academy of Higher Education, Manipal, India; 2 Department of Orthopedics, Kasturba Medical College Mangalore, Manipal Academy of Higher Education, Manipal, India; 3 Department of Radiology, Kasturba Medical College Mangalore, Manipal Academy of Higher Education, Manipal, India; 4 College of Clinical and Rehabilitation Health Sciences, East Tennessee State University, Johnson City, TN, USA

**Keywords:** Knee Osteoarthritis, Core muscle, Strength training, Rehabilitation, Exercise Therapy.

## Abstract

**Background:**

Knee osteoarthritis (KOA) is a prevalent condition. Recent research on people with KOA has addressed kinetic chain and core muscle contribution in disease progression.

**Objective:**

This study aims to assess the efficacy of including the CARE -KOA© regimen and evaluate its effect on pain, patient-reported functional outcomes, physical function tests, knee strength, and core endurance.

**Methods/Design:**

15 patients above 45 were recruited and underwent a 4-week CARE -KOA© program with 12 supervised sessions over four weeks. Pre- and Post-exercise assessment included evaluating the primary outcome pain using the VAS scale and the patient-reported outcome using the KOOS scale. The secondary outcomes, knee muscle strength, core endurance, and the physical function tests, i.e., the stair climb test, the sit-to-stand test, the 40m fast-paced walking test, and the timed up-and-go test, were also evaluated. The data collected in the study was analyzed using the statistical software JAMOVI. *p* < 0.05 was significant.

**Results:**

Notably, all the parameters examined exhibited a statistically significant difference between their pre-intervention and post-intervention values, except the knee muscle strength in the flexors of the affected knee and extensors of the unaffected knee.

**Conclusion:**

Patients who completed a 4-week supervised CARE -KOA© program alongside routine rehabilitation experienced reduced pain and improved outcomes. This approach aims to address biomechanical issues and positively impacts pain mechanisms.

**Study Trial Registration:**

CTRI/2023/07/05480 on 05/07/2024 https://ctri.nic.in/Clinicaltrials/regtrial.php?modid=1&compid=19&EncHid=69416.70327

**Copy right registration:** L – 158197/2024

## Introduction

Knee osteoarthritis (KOA) is a widely prevalent condition resulting in persistent disability. The combined prevalence of KOA was 16% for individuals aged 15 and above, escalating to 22.9% in the demographic aged 40 and beyond worldwide. ^1^ In India, up to 28.7% of people who have knee pain and signs of KOA report being unable to perform everyday tasks. ^2, 3^

Current international guidelines, including those from the Osteoarthritis Research Society International (OARSI), recommend structured, land-based exercise and patient education as core treatments for knee osteoarthritis, with or without weight management, due to their proven efficacy in reducing pain and improving function. ^4^

Conservative treatment for KOA emphasizes decreasing the compressive forces on the joint. Exercise therapy is a cornerstone in managing knee osteoarthritis, with robust evidence demonstrating its efficacy in reducing pain and improving function. A comprehensive systematic review and meta-analysis of 54 randomized controlled trials found that land-based therapeutic exercise provides moderate short-term benefits in pain reduction and functional improvement, with effects sustained for at least 2–6 months post-intervention. ^5^ It might be accomplished by strengthening the lower extremity's muscle strength, particularly the quadriceps muscle, which not only influences the initiation and progression of the disease but also plays an integral part in functional limitations in those with KOA. ^6– 9^

Recent studies highlight the role of proximal muscles in the role of disease progression. Training the core can enhance trunk, pelvic, hip, and knee stability and coordination by stimulating the periarticular muscles of the knee and the lumbopelvic hip complex. ^10^ Initial implications of the proximal contributions and the kinetic chains have recently been investigated in individuals with KOA, and a link between KOA and poor core has been seen as a plausible avenue contributing to the progression of the disease. ^10^

Compared to routine rehabilitation alone, 12 weeks of generalized core exercise with a routine rehabilitation program was superior and more efficient in reducing pain in patients who had KOA. ^11^ It has been demonstrated that various periarticular muscle exercise regimens and pharmaceutical treatments effectively minimize discomfort and improve physical function. The muscles that stabilize the knee joint are known to atrophy and lose strength because of KOA. ^12^

As a result, this study explored the effectiveness of incorporating the CARE-KOA© program and evaluated its efficacy on pain, patient-reported functional outcomes, physical function tests, knee strength, and core endurance in patients diagnosed with KOA, alongside, exploring the feasibility of conducting a future randomized controlled trial, to assess the long-term effect and adherence of the patient population to the program.

**Study Trial Registration**: CTRI/2023/07/05480 on 05/07/2024 https://ctri.nic.in/Clinicaltrials/regtrial.php?modid=1&compid=19&EncHid=69416.70327

## Methods

### Study design

A prospective, single group, pre- and post-study that was presented and approved by the Kasturba Medical College Mangaluru Institutional Ethics Committee (IECKMCMLR05/2023/206) on 18/5/2023 and recruitment was carried out from June 18 2023. The study was then registered with the Clinical Trials Registry India (CTRI) (CTRI/2023/07/054805). The study was chosen to test the effectiveness of using the CARE-KOA© and to evaluate its effects on the desired outcomes in patients diagnosed with KOA. The reporting of this study was in line with the Consolidated Standards of Reporting Trials (CONSORT). Our interest in this early investigation was to maximize the exploration of study methods and performance outcomes in patients diagnosed with KOA. 4- weeks was selected as the program length for this study. Informed consent was signed by all the participants included in the study, and all ethical standards defined by the Helsinki declaration were abided by.

### Participant and setting

The study was carried out in the hospital settings of Kasturba Medical College Mangalore.

Patients with a medical diagnosis of KOA, referred by the orthopaedic surgeon to the Physical Therapy Department, were included. The diagnosis was made by an orthopaedic surgeon specializing in knee conditions, based on the patient’s medical history (knee pain with crepitus during active motion, morning stiffness or bony enlargement, age, and a physical examination to rule out other causes of knee pain), along with radiographic imaging showing a K-L grade of 1-3.

Patients with severe KOA, in whom knee replacement is indicated, and those with a history of hip OA, lower limb joint replacement, inflammatory arthritis, spine surgery, lower limb surgery, or corticoid injection within the past three months, were excluded.

### Procedure

The purpose of the study was explained to the patients before enrolment and a written informed consent was obtained. Pain using the Visual Analog Scale (VAS), Patient-reported Outcomes using the Knee Injury and Osteoarthritis Outcome Score (KOOS) and Physical function test i.e., 30 seconds to stand test (30STS), 40 m fast-paced walking test (40MFPW), the stair climb test (SCT) and the timed up and go test (TUG), along with knee muscle strength, core muscle strength, and endurance was recorded by a qualified assessor who was blinded to the intervention and had adequate knowledge of the assessment tool, following a standardized procedure that has been previously described and published in the literature. ^13^

Demographic and baseline data were noted on the day the patient was referred for physiotherapy and then at four weeks post-intervention. Exercises were progressed based on the CARE -KOA© program and each exercise session lasted for an hour and included a 10-minute warmup session. ^21^

### Sample size determination and statistical analysis

A target sample size of n = 15 participants was achieved based on a pragmatic approach in the context of the study. Once eligibility was assessed and participants signed the informed consent form, they underwent the first evaluation and started with the four-week exercise routine.

The JAMOVI software was utilized to conduct statistical analysis, baseline data analysis, and non-parametric testing (Wilcoxon signed rank test) for within-group analysis. p < 0.05 was statistically significant.

### Participant flow

**Figure 1.**
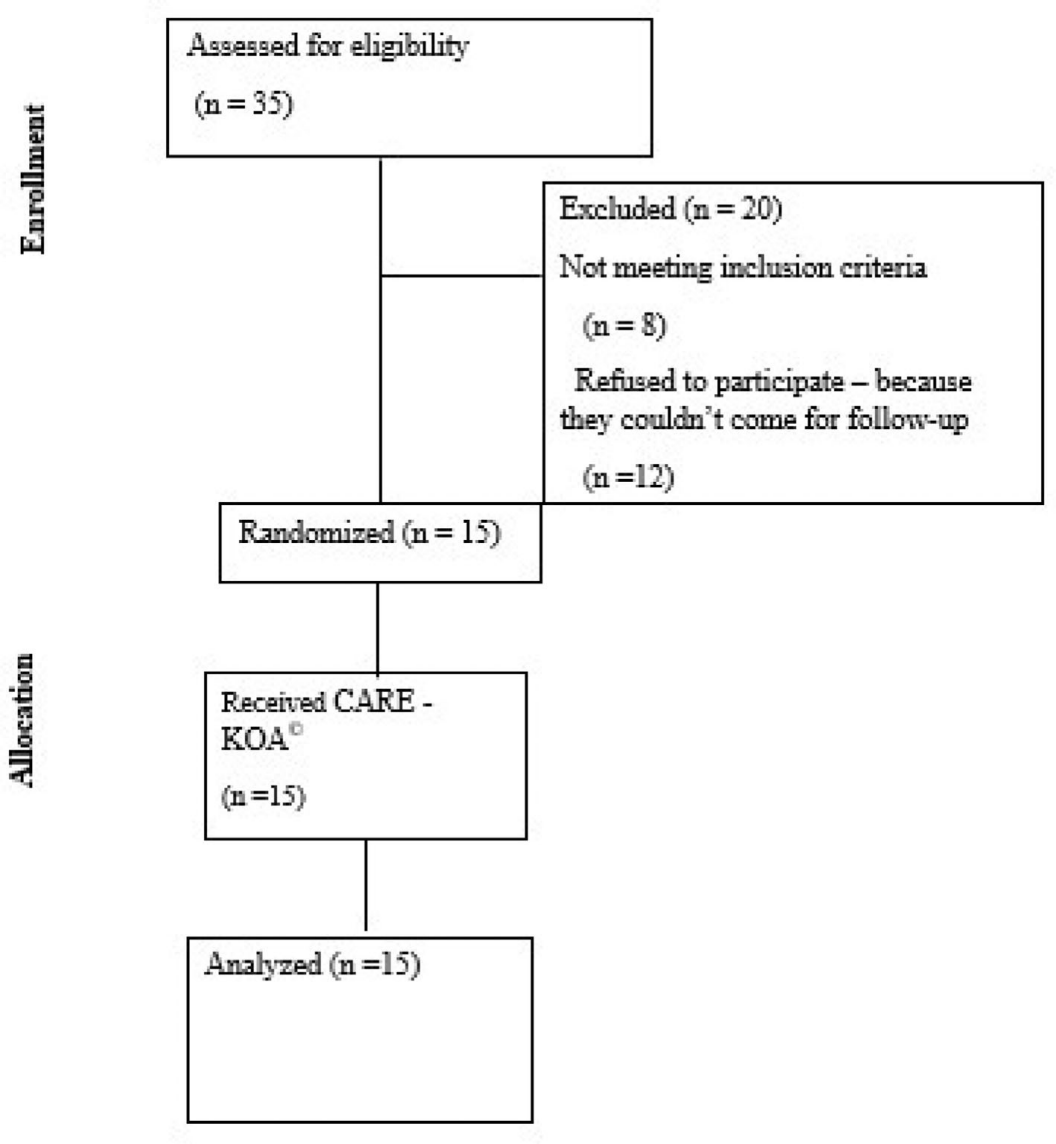
CONSORT flow diagram.

The patient enrolment in the study is depicted in Figure 1 flow diagram ( Figure 1). Fifteen participants were included after screening 35 patients with KOA.

## Results

15 participants over the age of 50 years were recruited ( Table 1) shows the descriptive data of the included participants.

**Table 1.** Descriptive data for participants (n = 15).

Variable	Median	IQR
Age (year)	54	50.0-56.5
Height (cm)	159	155-166
Weight (kg)	72	69.0-77.0
BMI	26.1	25.1-31.2

BMI, body mass index.

After four weeks of intervention, the patients reported less pain both at rest and during activity, along with significant improvements in functional and patient-reported outcomes as shown in Table 2. The muscle strength of the flexors in the unaffected knee and the extensors of the affected knee demonstrated statistically significant gains ( Table 2); however, muscle strength in the flexors of the affected knee and the extensors of the unaffected knee (p = 0.104 and p = 0.433, respectively) did not show a statistically significant difference.

**Table 2.** Comparison of pre- and post-intervention results on functional and clinical outcomes.

Measure	Baseline Mean ± SD	Post Mean ± SD	p-value	Effect Size (Cohen's d)
Pain at Rest (VR) (cm)	1.53 ± 1.30	0.40 ± 0.74	0.0006 *	1.07
Pain During Activity (VA) (cm)	6.33 ± 1.29	3.87 ± 1.51	0.000001 *	1.76
KOOS Pain (KP)	51.93 ± 12.72	62.27 ± 12.31	0.00003 *	-0.83
KOOS Symptoms (KS)	55.93 ± 10.89	64.33 ± 8.82	0.0006 *	-0.85
KOOS ADL (KADL)	51.60 ± 11.29	64.00 ± 9.43	0.0000009 *	-1.19
KOOS QOL (KQOL)	41.27 ± 9.04	50.47 ± 9.80	0.00017 *	-0.98
30-Second Sit-to-Stand (30STS) (reps)	8.13 ± 3.04	9.00 ± 2.59	0.050 *	-0.31
40-Meter Fast-paced walking test (40-MFPW) (m/sec)	1.00 ± 0.28	0.96 ± 0.25	0.044 *	-0.15
Stair Climb Test (SCT) (sec)	33.67 ± 10.10	30.73 ± 7.03	0.031 *	0.34
Timed Up and Go Test (TUG) (sec)	33.33 ± 18.68	31.07 ± 17.36	0.006 *	0.13
Endurance Test (ET) (sec)	17.73 ± 8.16	19.67 ± 9.80	0.027 *	0.21
Muscle Strength - Flexors Unaffected (MSFUA) (kg)	4.80 ± 1.93	5.20 ± 1.90	0.028 *	-0.21
Muscle Strength - Extensors Affected (MSEA) (kg)	5.07 ± 2.37	5.53 ± 2.59	0.013 *	-0.19
Muscle Strength - Flexors Affected (MSFA) (kg)	4.00 ± 1.93	4.27 ± 2.05	0.104	-0.13
Muscle Strength - Extensors Unaffected (MSEUA) (kg)	6.53 ± 3.62	6.67 ± 3.70	0.433	-0.04

**Abbreviations** - KOOS Pain ( **KP**); KOOS Symptom ( **KS**); KOOS Activities of Daily Living ( **KADL**); KOOS Sports and Recreation ( **KREC**); KOOS Quality of Life ( **KQOL**); 30-Second Sit to Stand ( **30STS**); 40-Second Fast-Paced Walking ( **40PFW**); Stair Climb Test ( **SCT**); Timed Up and Go Test ( **TUG**); Endurance Test ( **ET**); Muscle Strength Flexors Unaffected ( **MSFUA**); Muscle Strength Extensors Affected ( **MSEA**).

* p<0.05 was statistically significant.

## Discussion

The findings of this study provide increasing evidence that integrating the CARE-KOA© program into rehabilitation significantly enhances functional outcomes and reduces pain. This preliminary study indicates a notable reduction in pain and an improvement in various functional outcomes, highlighting the potential of core activation exercises in modulating pain mechanisms beyond conventional rehabilitation strategies.

A statistically and clinically significant reduction in pain at rest and during activity (d = 1.07 and d-1.76) as measured by VAS highlights the efficiency of the CARE-KOA© program in modulating pain mechanisms. Pain in KOA is multifactorial and influenced by joint degeneration, altered loading patterns, and neuromuscular imbalances. The core musculature, particularly the transverse abdominus and multifidus, contribute to trunk stability and pelvic alignment, preventing excessive strain on the knee joint. ^10, 14^

One of the most significant results of this research is the clinically meaningful improvement in patient-reported outcomes, specifically pain reduction (KOOS Pain, d = -0.83), symptomatic relief (KOOS Symptoms, d = -0.85), functional capacity (KOOS-ADL, d = -1.19), and overall quality of life (KOOS-QOL, d = -0.98). These findings underscore the critical role of kinetic chain activation in addressing biomechanical deficits associated with knee osteoarthritis (KOA).

Unlike traditional rehabilitation methods that mainly focus on strengthening the quadriceps, this study emphasizes the importance of targeting core muscle activation to enhance knee joint stability, improve load distribution, and optimize movement efficiency. The improvement in pain reduction and functional capacity can be attributed to enhanced stability resulting from the involvement of core stabilizers in the exercise regimen, which likely plays a pivotal role in offloading knee joint stress, thereby reducing pain and improving function. Furthermore, the observed changes are consistent with prior research indicating that kinetic chain impairments in KOA extend beyond the knee, affecting proximal joint coordination and motor function control. ^10, 15^

Physical function tests demonstrated meaningful improvements across the parameters assessed. The SCT and the core endurance test showed a moderate effect size, (d = 0.34) indicating that patients experienced significant functional gains in core endurance and stair negotiation. Similarly, the 30 STS and the 40MFPW showed improvements with a small effect size (d = -0.31 and d = -0.15), indicating improved mobility and walking efficiency. Additionally, the TUG (d = 0.13) showed significant improvements, reflecting better reaction time and movement efficiency. These gains indicate improved endurance, balance, and mobility, all of which are necessary for performing everyday tasks that retain independence and improve dynamic balance. ^16^ The core endurance test (ET, d = 0.21) demonstrated a small effect size, highlighting the role of core endurance in functional performance. Our findings align with previous studies, highlighting that exercises lead to improvements in mobility and functional performance, ultimately helping those affected to maintain their independence, reduce risk to all, and enhance overall quality of life. ^15^

Our findings are consistent with previous high-quality evidence, including a large systematic review and meta-analysis, which concluded that land-based exercise interventions yield significant improvements in pain, physical function, and quality of life in individuals with knee OA. The sustained benefits observed in our study further support the integration of structured exercise programs as a fundamental component of knee OA management. ^4^ The intervention’s comprehensive strategy, which emphasized strengthening core muscles, likely contributed to improved overall stability, movement mechanics, and functional capabilities For the trunk and pelvis to remain stable and to preserve joint loading patterns and lower limb biomechanics, the core muscles must provide dynamic stability. ^10, 14^ Strengthening exercises for the core muscles can help distribute forces more evenly across all joints, reducing the mechanical strain on the injured knee and relieving discomfort. Additionally, improved trunk stability and alignment may have improved joint biomechanics and reduced pain and discomfort during weight bearing. ^14^

Notably, while improvements were seen in most indicators, there were no appreciable gains in the strength of the knee flexors and extensors of the affected and unaffected knee respectively. This might be explained by the relatively brief intervention period of exercises and the nature of the muscles to adapt to load over some time, implying that longer duration and frequency of exercises are required to observe plausible strength changes. ^17, 18^

The exercise regime incorporated in our study was in line with the previous studies ^16, 19^ where a more holistic core exercise program was incorporated, whereas the current study targeted the TA and multifidus, which are considered the prime stabilizer muscles of the core. ^20^ The patients tolerated the exercises well; no adverse events were noted during the 4-week intervention. To provide a more comprehensive knowledge of the intervention's benefits, our study also examined various outcomes, such as patient-reported measures, physical function tests, knee strength, and core endurance.

Long-term follow-up examinations are necessary to assess whether outcomes may be sustained beyond the short duration of the intervention. The study establishes the foundation for further research by offering insightful information on the possible advantages of including the CARE -KOA© program in treating KOA.

Given the improvements in pain management, patient-reported outcomes, and functional performance, a complete strategy incorporating focused core exercises appears promising for treating KOA patients. A limitation noted in this study was the exclusion of patients who could not attend follow-up appointments. This highlights that access to the physiotherapy department and the ability to participate in follow-ups were essential criteria for participation in the exercise program. This ensured adherence to the program.

Our findings suggest that the CARE-KOA© program may benefit individuals with KOA by emphasizing proximal stability and biomechanical efficiency. By integrating core activation strategies into routine rehabilitation, clinicians can offer an evidence-based intervention that enhances the mobility, independence, and overall quality of life of individuals with KOA.

## Conclusion

This study evaluated the effectiveness of the CARE-KOA© program in patients with knee osteoarthritis, specifically assessing its impact on pain, patient-reported functional outcomes, physical function, knee strength, and core endurance. Following the 4-week supervised intervention, participants experienced significant reductions in pain at rest and during activity, as well as notable improvements in patient-reported outcomes (KOOS), physical function tests (sit-to-stand, stair climb, walking, and timed up-and-go), and core endurance. However, increases in knee muscle strength were observed primarily in the flexors of the unaffected knee and extensors of the affected knee, while other muscle groups did not show statistically significant changes.

These findings suggest that incorporating a structured core activation and rehabilitation program can enhance multiple dimensions of knee osteoarthritis management, particularly pain, function, and core stability. The short intervention duration may have limited the extent of strength gains, indicating the need for longer-term studies to assess sustained effects and optimize exercise protocols.

Future research should include larger sample sizes, control groups, and extended follow-up to confirm these results and further explore the long-term benefits and adherence to the CARE-KOA© program. Overall, this preliminary evidence supports the integration of core-focused rehabilitation into standard care for patients with knee osteoarthritis.

## Ethics and consent

Ethical Approval and Consent to Participate – The independent institutional ethical committee has approved the study and has the ethical number (IECKMCMLR05/2023/206). The study was then registered with the Clinical Trials Registry India (CTRI) (CTRI/2023/07/054805). Kasturba Medical College Mangaluru Institutional Ethics Committee (IECKMCMLR05/2023/206) on 18/5/2023 and recruitment was carried out from June 18 2023.

Consent for publication - All participants included in the study signed a written informed consent form, which was approved by the institutional ethics committee.

## Data Availability

Repository name: OSF The project contains the following underlying data:
• CARE-KOA PROTOCOL CARE -KOA https://doi.org/10.17605/OSF.IO/5E34P
• The data in this study has been registered on the OSF database https://doi.org/10.17605/OSF.IO/R4MQD ^21^ Data are available under the terms of the Creative Commons Attribution 4.0 International license (CC-BY 4.0). Repository name: OSF https://doi.org/10.17605/OSF.IO/R4MQD
