# Effectiveness Of The Core Activation And Rehabilitation Exercises For Knee Osteoarthritis - Program (CARE -KOA ^©^) Among Patients Diagnosed With Knee Osteoarthritis.

**DOI:** 10.12688/f1000research.163321.4

**Published:** 2025-07-21

**Authors:** Dias Tina Thomas, Charu Eapen, Atmananda S Hegde, Ajit R. Mahale, Prajwal Prabhudev Mane, Saurabh Mehta

**Affiliations:** 1 Department of Physiotherapy, Kasturba Medical College Mangalore, Manipal Academy of Higher Education, Manipal, India; 2 Department of Orthopedics, Kasturba Medical College Mangalore, Manipal Academy of Higher Education, Manipal, India; 3 Department of Radiology, Kasturba Medical College Mangalore, Manipal Academy of Higher Education, Manipal, India; 4 College of Clinical and Rehabilitation Health Sciences, East Tennessee State University, Johnson City, TN, USA

**Keywords:** Knee Osteoarthritis, Core muscle, Strength training, Rehabilitation, Exercise Therapy.

## Abstract

**Background:**

Knee osteoarthritis (KOA) is a prevalent condition. Recent research highlights the role of kinetic chain and core muscle involvement in disease progression, yet evidence for structured core activation protocols such as CARE-KOA© remains limited.This study addresses this gap by evaluating the effectiveness of CARE-KOA©, which specifically targets proximal stability and biomechanical deficits in KOA, aiming to enhance pain, function, and core endurance beyond conventional approaches.

**Methods:**

This prospective single-group pre-post study assessed the effect of a Participants underwent a 4-week CARE-KOA© regimen (12 supervised sessions, each including a 10-minute warm-up and core-focused exercises). Pre- and post-intervention assessments included pain (Visual Analog Scale, VAS), patient-reported outcomes (Knee Injury and Osteoarthritis Outcome Score, KOOS), physical function tests (30-second sit-to-stand, 40-meter fast-paced walk, stair climb, timed up-and-go), and knee muscle strength and core endurance. Statistical analysis was performed using non-parametric tests in JAMOVI.

**Results:**

Significant improvements were observed in pain at rest (mean change: 1.13 cm, p = 0.0006, d = 1.07) and during activity (mean change: 2.46 cm, p = 0.000001, d = 1.76), patient-reported outcomes (KOOS Pain: p = 0.00003, d = -0.83; KOOS ADL: p = 0.0000009, d = -1.19), and core endurance (p = 0.027, d = 0.21). Physical function tests also improved (stair climb: p = 0.031, d = 0.34; timed up-and-go: p = 0.006, d = 0.13). Muscle strength gains were significant in flexors of the unaffected knee and extensors of the affected knee (p < 0.05), while other muscle groups showed no significant change.

**Conclusion:**

The CARE-KOA© program led to clinically meaningful improvements in pain, function, and core endurance, highlighting the value of core activation strategies in KOA management. Future research with larger samples and longer follow-up is warranted to confirm these benefits and optimize exercise protocols.

**Study Trial Registration:**

CTRI/2023/07/05480 on 05/07/2024 https://ctri.nic.in/Clinicaltrials/regtrial.php?modid=1&compid=19&EncHid=69416.70327

**Copy right registration:** L – 158197/2024

## Introduction

Knee osteoarthritis (KOA) is a widely prevalent condition resulting in persistent disability. The combined prevalence of KOA was 16% for individuals aged 15 and above, escalating to 22.9% in the demographic aged 40 and beyond worldwide. ^1^ In India, up to 28.7% of people who have knee pain and signs of KOA report being unable to perform everyday tasks. ^2, 3^

Current international guidelines, including those from the Osteoarthritis Research Society International (OARSI), recommend structured, land-based exercise and patient education as core treatments for knee osteoarthritis, with or without weight management, due to their proven efficacy in reducing pain and improving function. ^4^

Conservative treatment for KOA emphasizes decreasing the compressive forces on the joint. Exercise therapy is a cornerstone in managing knee osteoarthritis, with robust evidence demonstrating its efficacy in reducing pain and improving function. A comprehensive systematic review and meta-analysis of 54 randomized controlled trials found that land-based therapeutic exercise provides moderate short-term benefits in pain reduction and functional improvement, with effects sustained for at least 2–6 months post-intervention. ^5^ It might be accomplished by strengthening the lower extremity’s muscle strength, particularly the quadriceps muscle, which not only influences the initiation and progression of the disease but also plays an integral part in functional limitations in those with KOA. ^6– 9^

Recent studies highlight the role of proximal muscles in disease progression. Training the core can enhance trunk, pelvic, hip, and knee stability and coordination by stimulating the periarticular muscles of the knee and the lumbopelvic hip complex. ^10^ Initial implications of the proximal contributions and the kinetic chains have recently been investigated in individuals with KOA, and a link between KOA and poor core has been seen as a plausible avenue contributing to the progression of the disease. ^10^

Compared to routine rehabilitation alone, 12 weeks of generalized core exercise with a routine rehabilitation program was superior and more efficient in reducing pain in patients who had KOA. ^11^ It has been demonstrated that various periarticular muscle exercise regimens and pharmaceutical treatments effectively minimize discomfort and improve physical function. The muscles that stabilize the knee joint are known to atrophy and lose strength because of KOA. ^12^

The Core Activation and Rehabilitation Exercises for Knee Osteoarthritis (CARE-KOA©) protocol, developed and copyrighted (L–158197/2024) is a structured, supervised exercise regimen that specifically addresses proximal stability and biomechanical deficits in KOA. This protocol differs from traditional approaches by emphasizing core muscle activation—particularly the transverse abdominis and multifidus, to enhance trunk and pelvic stability, thereby reducing excessive strain on the knee joint and improving overall movement efficiency. ^13^

Despite robust evidence supporting exercise in KOA, the literature on the efficacy of core-focused protocols remains limited. A systematic review by Fransen et al. ^5^ (BJSM, 2015) and OARSI guidelines (Bannuru et al., 2019) ^4^ underscore the benefits of structured exercise but also highlight the need for innovative approaches targeting kinetic chain impairments.

As a result, this study explored the effectiveness of incorporating the CARE-KOA© program and evaluated its efficacy on pain, patient-reported functional outcomes, physical function tests, knee strength, and core endurance in patients diagnosed with KOA, alongside, exploring the feasibility of conducting a future randomized controlled trial, to assess the long-term effect and adherence of the patient population to the program.

**Study Trial Registration**: CTRI/2023/07/05480 on 05/07/2024 https://ctri.nic.in/Clinicaltrials/regtrial.php?modid=1&compid=19&EncHid=69416.70327

## Methods

### Study design

A prospective, single group, pre- and post-study that was presented and approved by the Kasturba Medical College Mangaluru Institutional Ethics Committee (IECKMCMLR05/2023/206) on 18/5/2023 and recruitment was carried out from June 18 2023. The study was then registered with the Clinical Trials Registry India (CTRI) (CTRI/2023/07/054805). The study was chosen to test the effectiveness of using the CARE-KOA© and to evaluate its effects on the desired outcomes in patients diagnosed with KOA. The reporting of this study was in line with the Consolidated Standards of Reporting Trials (CONSORT). Our interest in this early investigation was to maximize the exploration of study methods and performance outcomes in patients diagnosed with KOA. 4- weeks was selected as the program length for this study. Informed consent was signed by all the participants included in the study, and all ethical standards defined by the Helsinki declaration were abided by.

### Participant and setting

The study was carried out in the hospital settings of Kasturba Medical College Mangalore.

Patients with a medical diagnosis of KOA, referred by the orthopaedic surgeon to the Physical Therapy Department, were included. The diagnosis was made by an orthopaedic surgeon specializing in knee conditions, based on the patient’s medical history (knee pain with crepitus during active motion, morning stiffness or bony enlargement, age, and a physical examination to rule out other causes of knee pain), along with radiographic imaging showing a K-L grade of 1-3.

Patients with severe KOA, in whom knee replacement is indicated, and those with a history of hip OA, lower limb joint replacement, inflammatory arthritis, spine surgery, lower limb surgery, or corticoid injection within the past three months, were excluded.

### Procedure

The purpose of the study was to explain the procedure to patients before enrollment, and a written informed consent was obtained. Demographic and baseline data were noted on the day the patient was referred for physiotherapy and then at four weeks post-intervention. All assessments were performed by a trained physiotherapist who was blinded to intervention status. Each outcome measure was assessed twice (pre- and post-intervention). Strength testing: three trials per muscle group; best value recorded. Functional and patient-reported tests: one trial per assessment point.

Pain was assessed using the Visual Analog Scale (VAS), a validated, self-reported measure ranging from 0 (no pain) to 10 (worst pain imaginable). Participants rated their pain at rest and during activity, both at baseline and after the intervention. The Visual Analog Scale (VAS) for pain demonstrates excellent test-retest reliability (ICC > 0.90) and high validity for assessing pain intensity. ^14^

The Knee Injury and Osteoarthritis Outcome Score (KOOS) was used to assess patient-reported pain, symptoms, activities of daily living, sports/recreation, and quality of life. Each subscale is scored from 0 to 100, with higher scores indicating better outcomes. The KOOS is widely used and validated for use in populations with knee osteoarthritis. The Knee Injury and Osteoarthritis Outcome Score (KOOS) is validated for knee osteoarthritis populations with strong reliability (ICC 0.75–0.95). ^15^

Physical function was evaluated using the following tests: 30-Second Sit-to-Stand Test (30STS): The number of times participants could rise from a chair and sit back down in 30 seconds was recorded, reflecting lower limb strength and functional mobility. ^16^

40-Meter Fast-Paced Walk Test (40MFPW): Participants walked 40 meters at their fastest safe speed, and the time taken was recorded to assess walking speed and endurance. ^17^

Stair Climb Test (SCT): The time required to ascend and descend a standard flight of stairs was measured, reflecting functional strength and mobility. ^18^

Timed Up-and-Go Test (TUG): Participants rose from a chair, walked 3 meters, turned, returned, and sat back down. The time taken was recorded to assess mobility, balance, and fall risk. Performance-based tests such as the 30-Second Sit-to-Stand Test, 40-Meter Fast-Paced Walk Test, Stair Climb Test, and Timed Up-and-Go Test all exhibit high reliability (ICC values ranging from 0.84 to 0.99) and validity for functional assessment. ^19^

Isometric strength of the knee flexors and extensors for both the affected and unaffected limbs was measured using a handheld dynamometer. Both affected and unaffected sides were assessed to evaluate asymmetry and compensatory adaptations. Strength was recorded in kilograms, and the best of three trials was used. Handheld dynamometry for knee strength assessment shows good reliability (ICC 0.82–0.97). Core muscle endurance was assessed using a prone plank test, where participants maintained a static trunk position for as long as possible. Time in seconds was recorded, reflecting endurance of the trunk musculature. The prone plank test for core endurance demonstrates high reliability (ICC 0.97). ^20^

All assessments were performed by a trained assessor who was blinded to the intervention status of the participants, following standardized protocols as previously described in the literature. ^13^

Exercises were progressed based on the CARE-KOA program, and each exercise session lasted an hour, including a 10-minute warm-up session.

The CARE-KOA© protocol was developed at the Department of Physiotherapy, Kasturba Medical College Mangalore, Manipal Academy of Higher Education, and is registered under copyright (L – 158197/2024). The protocol was specifically designed to address core muscle activation and kinetic chain integration in individuals with knee osteoarthritis and has been previously published by the authors. ^13^

The program consisted of a 4-week supervised regimen, with 12 sessions (3 sessions/week), each lasting 60 minutes and including a 10-minute warm-up. Core activation exercises targeted the transverse abdominis and multifidus, with progressive loading and complexity based on participant tolerance and performance. The protocol included bridging, planks, dynamic exercises, and functional movements emphasizing core stability and pelvic alignment. Exercises were progressed by increasing repetition, holding time, resistance, or complexity, as tolerated by the participant. Progression was guided by a qualified physiotherapist who was blinded to the intervention ( Table 1).

**Figure 1.**
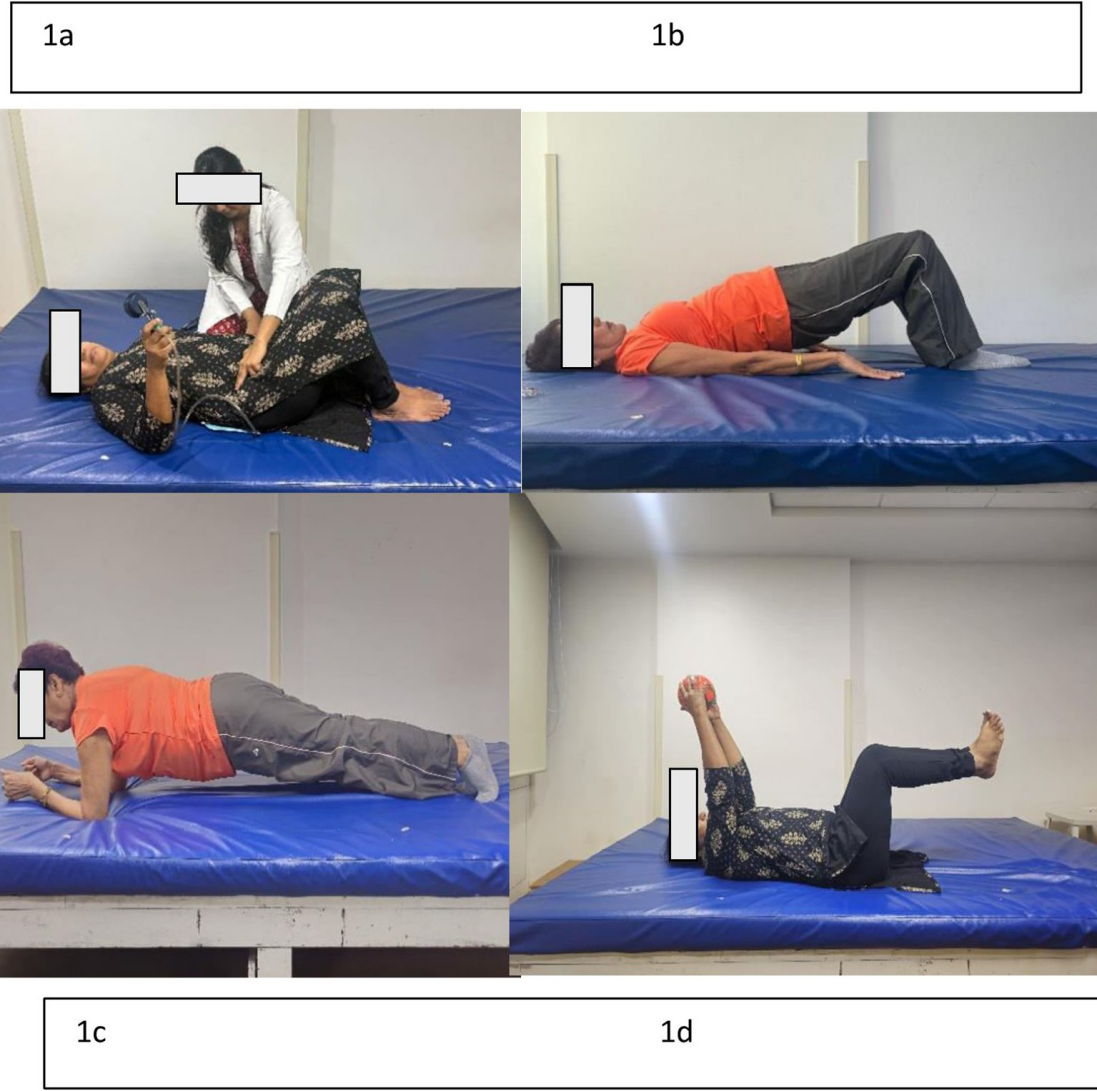
a) Transverse abdominus activation, b) Glute bridge, c) Prone bridge, d) Supine - over-head raises with knee bent.

A detailed description of the CARE-KOA protocol, including exercise types, progression, and session structure, is provided in the Supplementary Materials ( Tables 2 and 3). ^29^ All sessions were supervised by a physiotherapist trained in the CARE-KOA© protocol who was blinded to the study. No adverse events were reported during the intervention period ( Figure 1a-d).

**Table 3.** Routine rehabilitation protocol.

1 Static Quadriceps
2 SLR
3 Side-lying Hip abduction
4 Dynamic Quadriceps in sitting
5 Fast knee end-extensions
6 Small Swiss ball between the popliteal fossa and the wall
7 Low step height, light elastic band, straight forward steps
8 Wall squat
9 Forward Lunges

### Sample size determination and statistical analysis

A target sample size of n = 15 participants was achieved based on a pragmatic approach in the context of the study. Once eligibility was assessed and participants signed the informed consent form, they underwent the first evaluation and started with the four-week exercise routine.

The JAMOVI software was utilized to conduct statistical analysis, baseline data analysis, and non-parametric testing (Wilcoxon signed rank test) for within-group analysis. p < 0.05 was statistically significant.

### Participant flow

**Figure 2.**
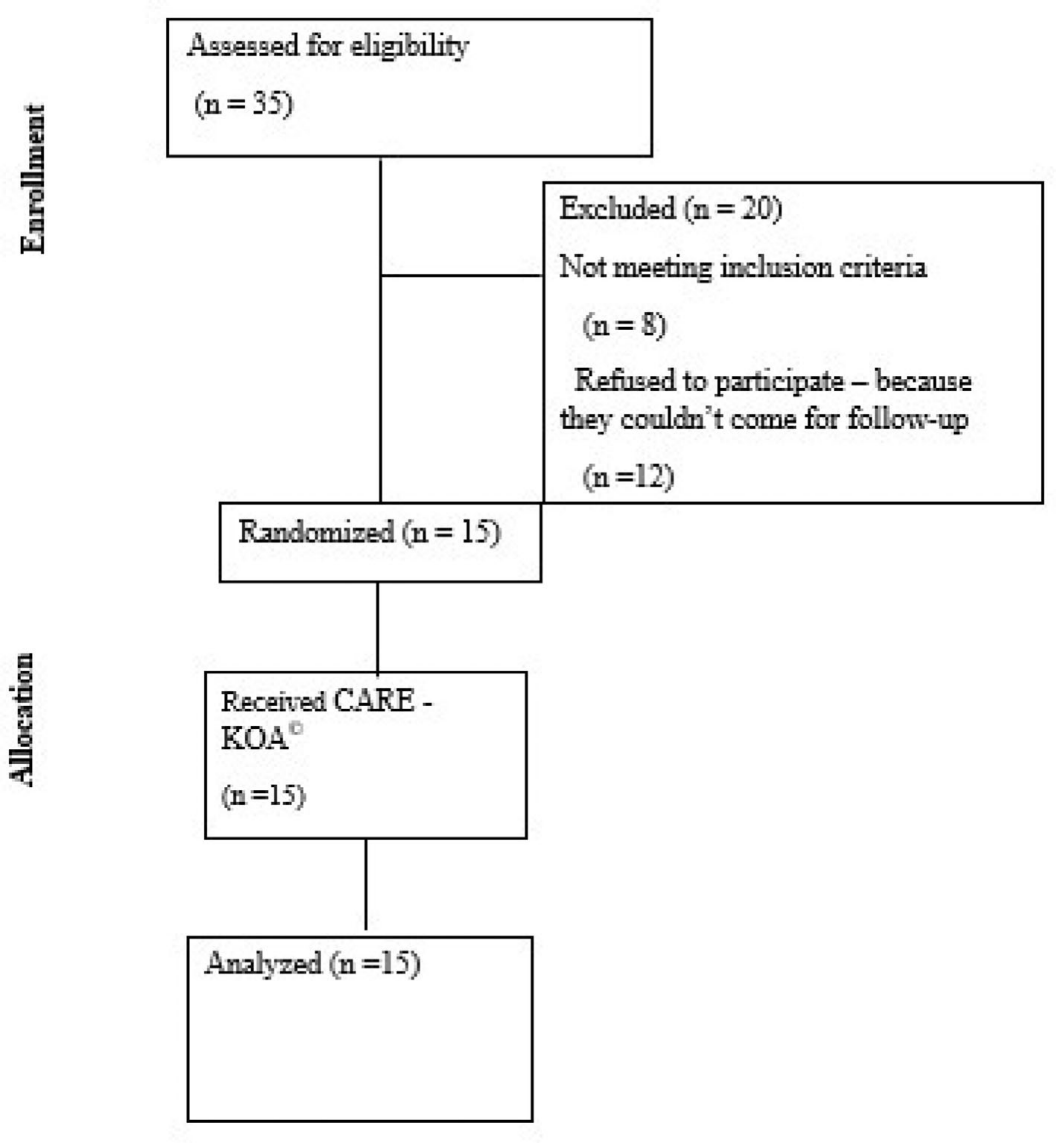
CONSORT flow diagram.

The patient enrolment in the study is depicted in flow diagram ( Figure 2). Fifteen participants were included after screening 35 patients with KOA.

## Results

15 participants over the age of 50 years were recruited ( Table 4) shows the descriptive data of the included participants. The sample included 9 females (60%) and 6 males (40%), with KOA grades distributed as Grade 1 (n = 3), Grade 2 (n = 10), and Grade 3 (n = 2). The majority of participants had right-sided knee involvement (73.3%).

**Table 4.** Descriptive data for participants (n = 15).

Variable	Median/IQR or n (%) (n = 15)
Age (year)	54 (50.0–56.5)
Gender	Female: 9 (60%), Male: 6 (40%)
KOA Grade	Grade 1: 3, Grade 2: 10, Grade 3: 2
Affected Side	Right: 11 (73.3%), Left: 4 (26.7%)
Height (cm)	159 (155–166)
Weight (kg)	72 (69.0–77.0)
BMI	26.1 (25.1–31.2)

BMI, body mass index.

**Table 1.** Exercise prescription

Component	Description
Frequency	3 sessions per week
Intensity	Progressive, based on participant tolerance
Time	60 minutes per session (including warm-up)
Type	Core activation + Routine Knee Rehabilitation
Progression	Increased repetitions, hold time, resistance, or complexity as tolerated
Volume	12 sessions over 4 weeks

Following the 4-week CARE-KOA© intervention, participants demonstrated statistically significant improvements across multiple outcome measures ( Table 5). Pain, as measured by the Visual Analog Scale (VAS), decreased from a mean of 1.53 cm at rest (SD 1.30) to 0.40 cm (SD 0.74; p = 0.0006, Cohen’s d = 1.07) and from 6.33 cm during activity (SD 1.29) to 3.87 cm (SD 1.51; p = 0.000001, Cohen’s d = 1.76), indicating large effect sizes and clinically meaningful reductions in both resting and activity-related pain.

**Table 5.** Comparison of pre- and post-intervention results on functional and clinical outcomes.

Measure	Baseline Mean ± SD	Post Mean ± SD	p-value	Effect Size (Cohen’s d)
Pain at Rest (VR) (cm)	1.53 ± 1.30	0.40 ± 0.74	0.0006 *	1.07
Pain During Activity (VA) (cm)	6.33 ± 1.29	3.87 ± 1.51	0.000001 *	1.76
KOOS Pain (KP)	51.93 ± 12.72	62.27 ± 12.31	0.00003 *	-0.83
KOOS Symptoms (KS)	55.93 ± 10.89	64.33 ± 8.82	0.0006 *	-0.85
KOOS ADL (KADL)	51.60 ± 11.29	64.00 ± 9.43	0.0000009 *	-1.19
KOOS QOL (KQOL)	41.27 ± 9.04	50.47 ± 9.80	0.00017 *	-0.98
30-Second Sit-to-Stand (30STS) (reps)	8.13 ± 3.04	9.00 ± 2.59	0.050 *	-0.31
40-Meter Fast-paced walking test (40-MFPW) (m/sec)	1.00 ± 0.28	0.96 ± 0.25	0.044 *	-0.15
Stair Climb Test (SCT) (sec)	33.67 ± 10.10	30.73 ± 7.03	0.031 *	0.34
Timed Up and Go Test (TUG) (sec)	33.33 ± 18.68	31.07 ± 17.36	0.006 *	0.13
Endurance Test (ET) (sec)	17.73 ± 8.16	19.67 ± 9.80	0.027 *	0.21
Muscle Strength - Flexors Unaffected (MSFUA) (kg)	4.80 ± 1.93	5.20 ± 1.90	0.028 *	-0.21
Muscle Strength - Extensors Affected (MSEA) (kg)	5.07 ± 2.37	5.53 ± 2.59	0.013 *	-0.19
Muscle Strength - Flexors Affected (MSFA) (kg)	4.00 ± 1.93	4.27 ± 2.05	0.104	-0.13
Muscle Strength - Extensors Unaffected (MSEUA) (kg)	6.53 ± 3.62	6.67 ± 3.70	0.433	-0.04

**Abbreviations** - KOOS Pain ( **KP**); KOOS Symptom ( **KS**); KOOS Activities of Daily Living ( **KADL**); KOOS Sports and Recreation ( **KREC**); KOOS Quality of Life ( **KQOL**); 30-Second Sit to Stand ( **30STS**); 40-Second Fast-Paced Walking ( **40PFW**); Stair Climb Test ( **SCT**); Timed Up and Go Test ( **TUG**); Endurance Test ( **ET**); Muscle Strength Flexors Unaffected ( **MSFUA**); Muscle Strength Extensors Affected ( **MSEA**).

* p<0.05 was statistically significant.

**Table 2.** Core exercises will be added to the routine rehabilitation protocol.

Order	Exercises	Muscle group activated	Reps	Sets	Progression criteria
1)	Core activation				
a)	Level 1: core activation - Draw in and hold for 10 seconds	Transverse Abdominus	8	2	When the participant can perform the exercise without exertion and keep the pelvis stable during movement, progress to the next level.
b)	Level 2 - Opposite LE on the mat; bent leg falls out	Transverse Abdominus	8	2	When the participant can perform the exercise without exertion and keep the pelvis stable during movement, progress to the next level.
c)	Level 3: A, B, or C - Opposite LE is on the table	Transverse Abdominus	8	2	When the participant can perform the exercise without exertion and keep the pelvis stable during movement, progress to the next level.
d)	Level 4: A, B, or C - old opposite LE @ 90 of hip flexion with UE	Transverse Abdominus	8	2	When the participant can perform the exercise without exertion and keep the pelvis stable during movement, progress to the next level.
e)	Level 5: A, B, or c - Hold opposite LE @ 90 of hip flexion (no UE assistance)	Transverse Abdominus	8	2	When the participant can perform the exercise without exertion and keep the pelvis stable during movement, progress to the next level.
f)	Level 6: A, B, or C - Bilateral LE movement	Transverse Abdominus	8	2	When the participant can perform the exercise without exertion and keep the pelvis stable during movement, progress to the next level.
2) a)	Bridge - hands at the side	Longissimus thoracisus + Lumbar Multifidus	8	2	When the participant can perform the exercise without exertion and keep the pelvis stable during movement, progress to the next level.
b)	Bridge - hands at the side and holds	Longissmus thoracisus + Lumbar Multifidus	8 * 10 seconds hold	2	When the participant can perform the exercise without exertion and keep the pelvis stable and hold 8 repetitions for 10 seconds progress to the next level.
c)	Bridge - hands across the chest	Longissmus thoracisus + Lumbar Multifidus	8	2	When the participant can perform the exercise without exertion and keep the pelvis stable during movement, progress to the next level.
d)	Bridge - hands across the chest with holds	Longissmus thoracisus + Lumbar Multifidus	8 * 10 seconds hold	2	When the participant can perform the exercise without exertion and keep the pelvis stable and hold 8 repetitions for 10 seconds progress to the next level.
e)	Unilateral Bridge	Longissmus thoracisus + Lumbar Multifidus	8	2	When the participant can perform the exercise without exertion and keep the pelvis stable during movement, progress to the next level.
f)	Unilateral Bridge - hands across the chest	Longissmus thoracisus + Lumbar Multifidus	8	2	When the participant can perform the exercise without exertion and keep the pelvis stable during movement, progress to the next level.
g)	Unilateral Bridge - hands across the chest with holds	Longissmus thoracisus + Lumbar Multifidus	8 * 10 seconds hold	2	When the participant can perform the exercise without exertion and keep the pelvis stable and hold 8 repetitions for 10 seconds progress to the next level.
h)	Bridge with both feet on the swiss ball	Longissmus thoracisus + Lumbar Multifidus	8 * 10 seconds hold	2	When the participant can perform the exercise without exertion and keep the pelvis stable and hold 8 repetitions for 10 seconds progress to the next level.
i)	Prone Bridge	External Oblique + Rectus Abdominus	2 * 30 seconds	2	When the participant can perform the exercise without distinct exertion and show good control of the trunk, the participant is progressed to the next level.
j)	Prone Bridge	External Oblique + Rectus Abdominus	2 * 1 minute	2	When the participant can perform the exercise without distinct exertion and show good control of the trunk, the participant is progressed to the next level.
3) a)	Supine - over-head raises with knee bent	Rectus abdominis + Internal obliques + External obliques + Transverse abdominis + Multifidus	8	2	When the participant can perform the exercise without exertion and keep the pelvis stable during movement, progress to the next level.
b)	Supine - holding a stability ball between your hands and knees - same side hand and leg movement	Rectus abdominis + Internal obliques + External obliques + Transverse abdominis + Multifidus	8	2	When the participant can perform the exercise without exertion and keep the pelvis stable during movement, progress to the next level.
c)	Supine Heel tap - Lower one foot at a time and tap the floor with your heel	Rectus abdominis + Internal obliques + External obliques + Transverse abdominis + Multifidus	8	2	When the participant can perform the exercise without exertion and keep the pelvis stable during movement, progress to the next level.
d)	Supine - Straight leg alternating hand and legs	Rectus abdominis + Internal obliques + External obliques + Transverse abdominis + Multifidus	8	2	When the participant can perform the exercise without exertion and keep the pelvis stable during movement, progress to the next level.

Patient-reported outcomes on the KOOS scale also improved significantly. The KOOS Pain subscale increased from 51.93 (SD 12.72) to 62.27 (SD 12.31; p = 0.00003, d = -0.83); the KOOS Symptoms subscale increased from 55.93 (SD 10.89) to 64.33 (SD 8.82; p = 0.0006, d = -0.85); the KOOS Activities of Daily Living (ADL) subscale increased from 51.60 (SD 11.29) to 64.00 (SD 9.43; p = 0.0000009, d = -1.19); and the KOOS Quality of Life subscale increased from 41.27 (SD 9.04) to 50.47 (SD 9.80; p = 0.00017, d = -0.98). These changes reflect substantial improvements in pain, symptoms, daily function, and overall quality of life following the intervention.

Physical function tests showed significant improvements. The 30-second sit-to-stand test (30STS) increased from 8.13 repetitions (SD 3.04) to 9.00 (SD 2.59; p = 0.050, d = -0.31), indicating enhanced lower limb strength and mobility. The 40-meter fast-paced walking test (40MFPW) showed a slight improvement, though with a small effect size (mean change not specified; p = 0.044, d = -0.15). The stair climb test (SCT) time decreased from 33.67 seconds (SD 10.10) to 30.73 seconds (SD 7.03; p = 0.031, d = 0.34), reflecting improved stair negotiation and functional mobility. The timed up-and-go test (TUG) also improved, with time decreasing from 33.33 seconds (SD 18.68) to 31.07 seconds (SD 17.36; p = 0.006, d = 0.13), suggesting better balance and movement efficiency. Core endurance, assessed by the endurance test (ET), increased from 17.73 seconds (SD 8.16) to 19.67 seconds (SD 9.80; p = 0.027, d = 0.21), indicating a small but significant improvement in core muscle endurance. *Muscle strength assessments revealed differential improvements by side.* The flexors of the unaffected knee showed significant gains (p = 0.028, d = -0.21), as did the extensors of the affected knee (p = 0.013, d = -0.19). However, muscle strength in the flexors of the affected knee and the extensors of the unaffected knee did not change significantly (p = 0.104 and p = 0.433, respectively). The unaffected side was assessed to monitor for compensatory changes and ensure no inadvertent deficits.

## Discussion

The findings of this study provide increasing evidence that integrating the CARE-KOA© program into rehabilitation significantly enhances functional outcomes and reduces pain. This preliminary study indicates a notable reduction in pain and an improvement in various functional outcomes, highlighting the potential of core activation exercises in modulating pain mechanisms beyond conventional rehabilitation strategies. The protocol’s emphasis on core muscle activation and kinetic chain integration distinguishes it from conventional rehabilitation, which typically focuses on quadriceps strengthening. By targeting the transverse abdominis and multifidus, CARE-KOA© promotes trunk and pelvic stability, thereby reducing excessive strain on the knee joint and improving overall movement efficiency. This approach addresses the kinetic chain, which is often overlooked in traditional protocols but is increasingly recognized as a key factor in KOA progression and functional limitation.

Compared to conventional protocols, CARE-KOA© offers several advantages. The integration of core activation exercises with functional movements leads to more holistic improvements in pain, function, and quality of life. Enhanced core stability improves load distribution across the knee joint, reduces pain, and optimizes movement patterns, resulting in greater improvements in daily activities and overall well-being. These findings align with recent literature, which highlights the importance of proximal stability and kinetic chain integration in the management of knee osteoarthritis.

A statistically and clinically significant reduction in pain at rest and during activity (d = 1.07 and d = -1.76) as measured by VAS highlights the efficiency of the CARE-KOA© program in modulating pain mechanisms. Pain in KOA is multifactorial and influenced by joint degeneration, altered loading patterns, and neuromuscular imbalances. The core musculature, particularly the transverse abdominus and multifidus, contribute to trunk stability and pelvic alignment, preventing excessive strain on the knee joint. ^10, 22^

One of the most significant results of this research is the clinically meaningful improvement in patient-reported outcomes, specifically pain reduction (KOOS Pain, d = -0.83), symptomatic relief (KOOS Symptoms, d = -0.85), functional capacity (KOOS-ADL, d = -1.19), and overall quality of life (KOOS-QOL, d = -0.98). These findings underscore the critical role of kinetic chain activation in addressing biomechanical deficits associated with knee osteoarthritis (KOA).

Unlike traditional rehabilitation methods that mainly focus on strengthening the quadriceps, this study emphasizes the importance of targeting core muscle activation to enhance knee joint stability, improve load distribution, and optimize movement efficiency. The improvement in pain reduction and functional capacity can be attributed to enhanced stability resulting from the involvement of core stabilizers in the exercise regimen, which likely plays a pivotal role in offloading knee joint stress, thereby reducing pain and improving function. Furthermore, the observed changes are consistent with prior research indicating that kinetic chain impairments in KOA extend beyond the knee, affecting proximal joint coordination and motor function control. ^10, 23^

Physical function tests demonstrated meaningful improvements across the parameters assessed. The SCT and the core endurance test showed a moderate effect size, (d = 0.34) indicating that patients experienced significant functional gains in core endurance and stair negotiation. Similarly, the 30 STS and the 40MFPW showed improvements with a small effect size (d = -0.31 and d = -0.15), indicating improved mobility and walking efficiency. Additionally, the TUG (d = 0.13) showed significant improvements, reflecting better reaction time and movement efficiency. These gains indicate improved endurance, balance, and mobility, all of which are necessary for performing everyday tasks that retain independence and improve dynamic balance. ^24^ The core endurance test (ET, d = 0.21) demonstrated a small effect size, highlighting the role of core endurance in functional performance. Our findings align with previous studies, highlighting that exercises lead to improvements in mobility and functional performance, ultimately helping those affected to maintain their independence, reduce risk to all, and enhance overall quality of life. ^23^

Our findings are consistent with previous high-quality evidence, including a large systematic review and meta-analysis, which concluded that land-based exercise interventions yield significant improvements in pain, physical function, and quality of life in individuals with knee OA. The sustained benefits observed in our study further support the integration of structured exercise programs as a fundamental component of knee OA management. ^4^ The intervention’s comprehensive strategy, which emphasized strengthening core muscles, likely contributed to improved overall stability, movement mechanics, and functional capabilities For the trunk and pelvis to remain stable and to preserve joint loading patterns and lower limb biomechanics, the core muscles must provide dynamic stability. ^10, 22^ Strengthening exercises for the core muscles can help distribute forces more evenly across all joints, reducing the mechanical strain on the injured knee and relieving discomfort. Additionally, improved trunk stability and alignment may have improved joint biomechanics and reduced pain and discomfort during weight bearing. ^22^

Notably, while improvements were seen in most indicators, there were no appreciable gains in the strength of the knee flexors and extensors of the affected and unaffected knee respectively. This might be explained by the relatively brief intervention period of exercises and the nature of the muscles to adapt to load over some time, implying that longer duration and frequency of exercises are required to observe plausible strength changes. ^25, 26^

Assessing both the affected and unaffected sides allowed for a comprehensive evaluation of asymmetry and compensatory adaptations associated with knee osteoarthritis. This approach is crucial, as patients often unconsciously rely more on the unaffected limb, potentially leading to overuse or strength deficits over time. Monitoring both sides ensured that the CARE-KOA© intervention promoted balanced improvements and did not inadvertently introduce new deficits. This comprehensive assessment supports the clinical goal of restoring symmetry and preventing secondary complications, ultimately enhancing patient outcomes and the applicability of the protocol in real-world settings.

The exercise regime incorporated in our study was in line with the previous studies ^24, 27^ where a more holistic core exercise program was incorporated, whereas the current study targeted the TA and multifidus, which are considered the prime stabilizer muscles of the core. ^28^ The patients tolerated the exercises well; no adverse events were noted during the 4-week intervention. To provide a more comprehensive knowledge of the intervention’s benefits, our study also examined various outcomes, such as patient-reported measures, physical function tests, knee strength, and core endurance.

Long-term follow-up examinations are necessary to assess whether outcomes may be sustained beyond the short duration of the intervention. The study establishes the foundation for further research by offering insightful information on the possible advantages of including the CARE -KOA© program in treating KOA.

Given the improvements in pain management, patient-reported outcomes, and functional performance, a complete strategy incorporating focused core exercises appears promising for treating KOA patients. A limitation noted in this study was the exclusion of patients who could not attend follow-up appointments. This highlights that access to the physiotherapy department and the ability to participate in follow-ups were essential criteria for participation in the exercise program. This ensured adherence to the program.

Our findings suggest that the CARE-KOA© program may benefit individuals with KOA by emphasizing proximal stability and biomechanical efficiency. By integrating core activation strategies into routine rehabilitation, clinicians can offer an evidence-based intervention that enhances the mobility, independence, and overall quality of life of individuals with KOA.

## Conclusion

This study evaluated the effectiveness of the CARE-KOA© program in patients with knee osteoarthritis, specifically assessing its impact on pain, patient-reported functional outcomes, physical function, knee strength, and core endurance. Following the 4-week supervised intervention, participants experienced significant reductions in pain at rest and during activity, as well as notable improvements in patient-reported outcomes (KOOS), physical function tests (sit-to-stand, stair climb, walking, and timed up-and-go), and core endurance. However, increases in knee muscle strength were observed primarily in the flexors of the unaffected knee and extensors of the affected knee, while other muscle groups did not show statistically significant changes.

These findings suggest that incorporating a structured core activation and rehabilitation program can enhance multiple dimensions of knee osteoarthritis management, particularly pain, function, and core stability. The short intervention duration may have limited the extent of strength gains, indicating the need for longer-term studies to assess sustained effects and optimize exercise protocols.

Future research should include larger sample sizes, control groups, and extended follow-up to confirm these results and further explore the long-term benefits and adherence to the CARE-KOA© program. Overall, this preliminary evidence supports the integration of core-focused rehabilitation into standard care for patients with knee osteoarthritis.

## Ethics and consent

Ethical Approval and Consent to Participate – The independent institutional ethical committee has approved the study and has the ethical number (IECKMCMLR05/2023/206). The study was then registered with the Clinical Trials Registry India (CTRI) (CTRI/2023/07/054805). Kasturba Medical College Mangaluru Institutional Ethics Committee (IECKMCMLR05/2023/206) on 18/5/2023 and recruitment was carried out from June 18 2023.

Consent for publication - All participants included in the study signed a written informed consent form, which was approved by the institutional ethics committee.

## Data Availability

Repository name: OSF The project contains the following underlying data:
• CARE-KOA PROTOCOL CARE -KOA https://doi.org/10.17605/OSF.IO/5E34P
• The data in this study has been registered on the OSF database https://doi.org/10.17605/OSF.IO/R4MQD ^29^ Data are available under the terms of the Creative Commons Attribution 4.0 International license (CC-BY 4.0). Repository name: OSF https://doi.org/10.17605/OSF.IO/R4MQD
