# Supplementary material for: Effectiveness Of The Core Activation And Rehabilitation Exercises For Knee Osteoarthritis - Program (CARE -KOA ©) Among Patients Diagnosed With Knee Osteoarthritis
Source: F1000Res. 2025 Jul 21;14:496. Originally published 2025 May 15. [Version 4] doi: 10.12688/f1000research.163321.4 (PMC12340487; doi:10.12688/f1000research.163321.4)
Supplement: Peer review history

# Reviewer report: Reviewer response for version 4

**DOI:** 10.5256/f1000research.184839.r399751

**Published:** 2025-08-11

**Authors:** Peeyoosha Gurudut, Aarti Welling

**Affiliations:** 1 Department of Orthopedic Physiotherapy, KLEU Institute of Physiotherapy, Belagavi, Karnataka, India; 2 Department of Orthopedic Physiotherapy, KLE Institute of Physiotherapy, KLE Academy of Higher Education & Research, Belagavi, Karnataka, India

I am glad to see the authors have incorporated all the changes and justified to all my comments. All the best with the journey.

Is the work clearly and accurately presented and does it cite the current literature?

Partly

If applicable, is the statistical analysis and its interpretation appropriate?

Yes

Are all the source data underlying the results available to ensure full reproducibility?

Partly

Is the study design appropriate and is the work technically sound?

Partly

Are the conclusions drawn adequately supported by the results?

Partly

Are sufficient details of methods and analysis provided to allow replication by others?

No

Reviewer Expertise:

Musculoskeletal, Physical therapy, Biomechanics, Kinesiotherapy, Physical treatments, aging, women

We confirm that we have read this submission and believe that we have an appropriate level of expertise to confirm that it is of an acceptable scientific standard.

# Reviewer report: Reviewer response for version 4

**DOI:** 10.5256/f1000research.184839.r399752

**Published:** 2025-08-11

**Authors:** Varun Naik

**Affiliations:** 1 KAHER Institute of Physiotherapy, Belagavi, Karnataka, India

Previous revisions are corrected.

Is the work clearly and accurately presented and does it cite the current literature?

Partly

If applicable, is the statistical analysis and its interpretation appropriate?

Partly

Are all the source data underlying the results available to ensure full reproducibility?

No

Is the study design appropriate and is the work technically sound?

Yes

Are the conclusions drawn adequately supported by the results?

Partly

Are sufficient details of methods and analysis provided to allow replication by others?

Partly

Reviewer Expertise:

Knee Rehabilitation

I confirm that I have read this submission and believe that I have an appropriate level of expertise to confirm that it is of an acceptable scientific standard.

# Reviewer report: Reviewer response for version 3

**DOI:** 10.5256/f1000research.183733.r393280

**Published:** 2025-07-08

**Authors:** Peeyoosha Gurudut

**Affiliations:** 1 Department of Orthopedic Physiotherapy, KLEU Institute of Physiotherapy, Belagavi, Karnataka, India

Who performed measurements and assessment? how many times each measure was assessed? how many trials. was the assessor blinded? qualification of assessor? what are psychometric properties of the outcomes as in ICC value validity or reliability of tests.. References for the outcomes are missing too. need to be added. 

 A tabular format for CARE-KOA protocol is needed for clarity on FITT-PV. Who gave intervention? authors seem to not have referred to CERT guidelines or checklist. Its emphasized for better readership and reproducibility of the copyright material. In the present format I cannot apply CARE protocol on my patients. 

 I still don't see images or figures for the protocol 

 Justify including affected and unaffected sides. were they compared? If not then why unaffected side was assessed?

 Other changes have been incorporated by the authors.

Is the work clearly and accurately presented and does it cite the current literature?

Partly

If applicable, is the statistical analysis and its interpretation appropriate?

Yes

Are all the source data underlying the results available to ensure full reproducibility?

Partly

Is the study design appropriate and is the work technically sound?

Partly

Are the conclusions drawn adequately supported by the results?

Partly

Are sufficient details of methods and analysis provided to allow replication by others?

No

Reviewer Expertise:

Musculoskeletal, Physical therapy, Biomechanics, Kinesiotherapy, Physical treatments, aging, women

I confirm that I have read this submission and believe that I have an appropriate level of expertise to confirm that it is of an acceptable scientific standard, however I have significant reservations, as outlined above.

# Reviewer report: Reviewer response for version 1

**DOI:** 10.5256/f1000research.179648.r387321

**Published:** 2025-06-14

**Authors:** Peeyoosha Gurudut

**Affiliations:** 1 Department of Orthopedic Physiotherapy, KLEU Institute of Physiotherapy, Belagavi, Karnataka, India

1. Abstract: Background needs more details on CARE protocol and gap in literature; Methods does not mention the study design. Results need to be more detailed with p values and effect sizes. Additionally results mentions affected and unaffected knees interpretation which is not explained in the methodology or table. Conclusion comments on biomechanical issues. which outcomes or tests assess the biomechanical problems is not clear. 

 2. Introduction: there is no clear introduction or overview given about this CARE  protocol which is copyrighted. or developer or previous studies and how this protocol is different from the conventional protocols. The rationale needs to be stronger that connects specifically to CARE as this is the highlight of your study

 3. Methodology: Considering your CARE protocol being highlight, this is very briefly explained. Intervention details of CARE protocol is entirely missing. this grossly affects reproducibility by other authors. no images are added or tables. no progression of the protocol. Who is the developer? can add these as annexure or table form and add few images. with references wherever needed/. Were both sides treated?

 4. Methodology: Even the outcome measures have not been explained in the detail. 

 5. Advise to authors to follow TiDiER checklist and CERT checklist to report the interventions and assessments. can easily find them on web browsers. 

 6. results: Gender wise distribution is missing. Even grades of KOA distribution is not added. side affected and occupation etc can be added in demographic details. 

 7. Affected and unaffected side details are not there in the table if at all compared which is mentioned in the abstract and text form.

 8. Results: more details need to be given in text of results for better readability and understanding. 

 Discussion: Most discussions only discuss results. The mechanism of the CARE protocol, how is it comparable to the conventional exercise protocol, and how is it better than other protocols need to be discussed. 

 Conclusion: This is not in line of the objective stated.

Is the work clearly and accurately presented and does it cite the current literature?

Partly

If applicable, is the statistical analysis and its interpretation appropriate?

Yes

Are all the source data underlying the results available to ensure full reproducibility?

Partly

Is the study design appropriate and is the work technically sound?

Partly

Are the conclusions drawn adequately supported by the results?

Partly

Are sufficient details of methods and analysis provided to allow replication by others?

No

Reviewer Expertise:

Musculoskeletal Physical therapy, Manual Therapy, Biomechanics or kinesiology, Kinesiotherapy, WOmen's health

I confirm that I have read this submission and believe that I have an appropriate level of expertise to confirm that it is of an acceptable scientific standard, however I have significant reservations, as outlined above.

# Reviewer report: Reviewer response for version 1

**DOI:** 10.5256/f1000research.179648.r387317

**Published:** 2025-06-05

**Authors:** Varun Naik

**Affiliations:** 1 KAHER Institute of Physiotherapy, Belagavi, Karnataka, India

1. The widely cited **( refer to 1 )** on exercise for knee OA is missing.

 2. No references from **OARSI (Osteoarthritis Research Society International)** guidelines are cited.

 3. Procedure of of intervention should be clearly mentioned for replication of the intervention.

 4. Conclusion not meeting the objectives of the study

Is the work clearly and accurately presented and does it cite the current literature?

Partly

If applicable, is the statistical analysis and its interpretation appropriate?

Partly

Are all the source data underlying the results available to ensure full reproducibility?

No

Is the study design appropriate and is the work technically sound?

Yes

Are the conclusions drawn adequately supported by the results?

Partly

Are sufficient details of methods and analysis provided to allow replication by others?

Partly

Reviewer Expertise:

Physiotherapy Interventions

I confirm that I have read this submission and believe that I have an appropriate level of expertise to confirm that it is of an acceptable scientific standard, however I have significant reservations, as outlined above.

## References

[1] : Exercise for osteoarthritis of the knee: a Cochrane systematic review. *British Journal of Sports Medicine* .2015;49(24) : 10.1136/bjsports-2015-095424 1554-1557 10.1136/bjsports-2015-095424 26405113
